# Effects of Dietary Fermented Black Garlic Powder Supplementation on Performance, Carcass Traits, Serum Parameters, Immune Response and Antioxidant Status of Japanese Male Quails

**DOI:** 10.1007/s11250-026-04871-8

**Published:** 2026-02-12

**Authors:** Behlül Sevim

**Affiliations:** Department of Food Processing, Technical Sciences Vocational Schools, Aksaray University, Aksaray, 68100 Türkiye

**Keywords:** Japanese quail, Fermented black garlic, Performance, Carcass, Antioxidants

## Abstract

This study investigated the effects of dietary supplementation with fermented black garlic powder on performance, carcass characteristics, serum parameters, immunological responses and antioxidant status in male Japanese quail. A total of 96 male Japanese quail aged  21-days, with similar initial body weights, were randomly allocated to four treatment groups with six replicates per treatment. The treatment diets were formulated by supplementing the basal diet with fermented black garlic powder at levels of  0, 2.5, 5.0 and 10.0 g/kg. Quails were fed *ad libitum* for 28 days. The results showed that fermented black garlic powder did not affect any of the performance parameters (*P* > 0.05). No differences were observed in any of the carcass characteristics; however, the relative heart weight decreased following fermented black garlic powder supplementation (*P<0.05*). Serum aspartat aminotransferase, cholesterol, low-density lipoprotein, total antioxidant status, and total oxidant status were not affected by dietary supplementation with fermented black garlic powder (*P>*0.05). In contrast, serum glucose, triglyceride, and immunoglobulin E concentrations decreased with increasing levels of fermented black garlic powder in the diet (*P*<0.01), whereas immunoglobulin G concentrations increased (*P*<0.05). The serum levels of alanin aminotransferase were highest in the control group and lowest in the group supplemented with 10 g/kg fermented black garlic powder (*P*<0.05). Serum levels of high-density lipoprotein were lowest and highest in quails supplemented with 2.5 and 10 g/kg fermented black garlic powder, respectively (*P*<0.05). Increasing the dietary levels of fermented black garlic powder increased the serum levels of immunoglobulin A (*P*<0.05). In conclusion, the result of this study that fermented black garlic powder has potential as an immune-enhancing feed additive in poultry diets, without negative affects on performance or carcass traits.

**Supplementary Information:**

The online version contains supplementary material available at https://doi.org/10.1007/s11250-026-04871-8.

## Introduction

The poultry industry has experienced remarkable growth in recent years, and thanks to advances in various scientific fields and extensive applied research efforts, poultry productivity has become prominent with its high efficiency (Kairalla et al. 2022). Feed additives have contributed to the sector’s advancement, and with the ban on antibiotics in recent years, the sector has sought alternative additives. Probiotics, prebiotics, organic acids, and medicinal and aromatic plants are promising alternatives.

Fermented feed additives are attracting attention as a rich source of currently investigated phytochemicals, bioactive compounds, and probiotics. The biological activities of these functional foods, such as anti-inflammatory and immunomodulatory functions, are largely due to their high antioxidant content (Shahbazi et al. 2021). Garlic (*Allium sativum* L.) is a common spice with numerous health benefits due to its various bioactive compounds, including organic sulfides, saponins, phenolic compounds, and polysaccharides (Mirzaei-Aghsaghali et al. 2012). Garlic, belonging to the Alliaceae family, contains at least 33 sulfur-containing compounds (Rana et al. 2011). Allicin (allyl 2-propenthiosulfinate or diallyl thiosulfinate) is the main bioactive compound. Black garlic is produced by storing white garlic in controlled environments at 45–90 °C and 50–90% relative humidity until it turns black. During production, Maillard and enzymatic reactions occur, leading to changes in the garlic’s physicochemical properties such as color, pH, dry matter, and nutritional value. Additionally, concentrations of total phenolic acids, flavonoids, 5-hydroxymethylfurfural, melanoidins, and thiosulfinates increase (Barido et al. 2022). Black garlic’s higher antioxidant activity compared to white garlic makes it preferred (Bayat and Petek 2024). While white and black garlic have similar effects such as immune-boosting, antitumor, and anti-inflammatory effects, the main antioxidant component in black garlic, S-allyl cysteine, is less toxic than other sulfur compounds (allicin and diallyl disulfide) in white garlic, its easy absorption, and rapid bioavailability make black garlic valuable (Erol and Ersus 2022). Furthermore, to benefit from the beneficial effects of garlic while reducing its disadvantages such as odor and digestibility, it is recommended to include fermented forms in poultry diets (Fathi et al. 2025). Although there are studies on garlic’s antimicrobial, antioxidant, and immune properties of garlic, research on the supplementation of black garlic in poultry diets remains insufficient Studies on garlic have reported that garlic powder improves performance in quails (Premavalli and Omprakash 2020), lowers cholesterol, improves antioxidant parameters (Mubarak, 2025), and improves carcass quality (Elsagheer et al. 2020).

The production of Japanese quail (*Coturnix coturnix* japonica) has recently gained importance due to their ease of management, short life cycle, and small size (Sarmiento-García et al., 2023). Male quails, raised for both meat and breeding, display higher carcass yields and greater breast and thigh + drumstick proportions than females (Tserveni-Gousi and Yannakopoulos, 1986; Sevim and Olgun 2021).

This study was designed based on the hypothesis that adding FBGP to the diet would boost the immune system in quails, as with white garlic, and would also improve performance and antioxidant properties. The aim of this study was to investigate the effects of adding FBGP to male quail diets on performance, carcass characteristics, serum characteristics, immunological parameters, and antioxidant properties.

## Materials and methods

### Ethical approval

The study was approved by the Ethics committee of Aksaray University (2025/1–2). In addition, all procedures have followed the European policy, for the safety of animals used for scientific research and other scientific purposes,   in accordance with European regulations for the protection of animals used for scientific purposes.

### Animals and diets

A total of 96 male Japanese quails (*Coturnix coturnix Japonica*) at the age of 21 days were used in the study, which lasted 28 days. In the experiment, quails were randomly distributed to four experimental groups and each group consisted of six replicates, each containing four male quails. The basal diet was prepared according to the nutritional levels recommended in NRC (1994) for Japanese quails, and experimental diets were prepared by adding 0, 2.5, 5.0 and 10.0 g/kg fermented black garlic powder (FBGP) to the basal diet. The ingredients of basal diet and nutrient composition are given Table 1. During the experiment, feed and water were given to quails as *ad libitum.* FBGP is supplied by a commercial company.

**Table 1:** Ingredients and nutrient content of the basal diet, as fed

Ingredients	%	Nutrients	%
Corn	49.50	Metabolizable energy, kcal/kg ME	2902
Soybean meal	43.50	Crude protein	23.97
Sunflower oil	3.20	Crude oil	5.43
Limestone	1.09	Crude fibre	3.81
Dicalcium phosphate	1.86	Calcium	1.01
Salt	0.35	Available phosphorus	0.50
Premix^1^	0.25	Lysine	1.34
DL-methionine	0.25	Methionine	0.53
Total	100	Methionine + cystine	1.00

^1^Premix provides 80 mg manganese (manganese oxide), 60 mg iron (iron carbonate), 5 mg copper (copper sulfate pentahydrate), 1 mg iodine, 0.15 mg selenium, 8800 IU vitamin A (trans-retinol acetate), 2200 IU vitamin D_3_ (cholecalciferol), 11 mg vitamin E (tocopherol), 44 mg nicotinic acid, 8.8 mg Cal-D-Pan, 4.4 mg vitamin B_2_ (riboflavin), 2.5 mg thiamine, 6.6 mg vitamin B_12_ (cyanocobalamin), 1 mg folic acid, 0.11 mg biotin, 220 mg choline to per kg of the diet

### Determination of performance parameters

In this study, initial and final body weights of the quails were determined by pen-based group weighing. Body weight gain (BWG) was calculated by subtracting the initial body weight (BW) values from the final BW values. The residual feeds were subtracted from the total feed given to the quails and the calculation was made considering the amount of feed intake (FI) by the dead quails. Feed intake (FI) was determined and expressed as g/day/quail and the feed conversion ratio (FCR) was determined from feed intake and body weight gain for each study period (4-week intervals) and for the total period of the experiment. 

### Carcass and organ traits

At the end of the experiment (49 days of age), one male Japanese quail randomly selected from each pen, weighed, and humanely euthanized via cervical dislocation. The weight of the hot carcass was recorded to calculate the hot carcass weight (HCW). The heart, liver, testis, and gizzard were weighed for each quail. The relative organ weights were calculated based on the dressed carcass weight of the quails and expressed as percentages.

### Blood serum analysis

At the end of the experiment (49 days of age), 3 mL of blood was collected from one male Japanese quail of similar body weight per replicate into 5 mL vacutainer tubes. The blood samples were centrifuged at 4000 rpm for 10 min to separate the serum. Glucose, aspartate aminotransferase (AST), alanine aminotransferase (ALT), and cholesterol concentrations in these serum were determined using an autoanalyzer (Biotecnica BT3000 Plus Chemistry Analyzer, Italy). Triglyceride, high-density lipoprotein (HDL), and low-density lipoprotein (LDL) were analyzed using a commercial kit (Beckman Coulter OSR). Immunoglobulin G (IgG), immunoglobulin E (IgE) and immunoglobulin A (IgA) analyses were performed turbidimetrically using the Abbot Architect Ci 8200 model device (Tietz 1995). Total Antioxidant status (TAS) and Total Oxidative Status (TOS) levels of serum were determined using commercial Rel Assay Diagnostic kits (Gaziantep, Turkey).

### Statistical analysis

Results were statistically evaluated for performance, carcass traits, meat quality, and bone biomechanical parameters. All available data were examined by one-way ANOVA with the SPSS 22.0 software package (SPSS Inc., Chicago, IL, USA). A P-value less than 0.05 was taken as statistically significant, while a P-value less than 0.10 was set as a trend. Orthogonal polynomial contrasts for determining the significance of linear and quadratic models were used. It was tested to describe the response of the variable to an increasingly high level of FBGP. 

## Results

Productive performance parameters, including initial body weight, final body weight, body weight gain, feed intake, feed conversion ratio of Japanese quail from 21 to 49 days of age presented in Table 2. Throughout the experiment, no significant differences were observed between the mean values of the treatment groups for initial body weight, final body weight, body weight gain, feed intake and feed conversion ratio (*P* > 0.05).

**Table 2:** Effects of different levels of fermented black garlic powder supplementation on performance traits of male Japanese quails (*n* = 96)

Traits	Fermented black garlic powder levels, g/kg				SEM	Anova	Linear	Quadratic
	0	2.5	5.0	10.0				
IBW (g)	89.66	89.66	88.33	90.33	0.531	0.629	0.893	0.371
FBW (g)	190.60	186.66	190.00	191.41	1.556	0.744	0.694	0.419
BWG (g)	100.93	97.00	101.66	101.08	1.570	0.735	0.730	0.614
FI (g/quail/day)	533.66	503.20	541.60	541.50	9.040	0.404	0.452	0.411
FCR	5.28	5.18	5.35	5.37	0.074	0.816	0.522	0.701

S.E.M.: Standard Error of the Mean. IBW: Initial Body Weight, FBW: Final Body Weight, BWG: Body Weight Gain, FI: Feed Intake, FCR: Feed Conversion Ratio

According to Table 3, the hot carcass weight, the liver, testes, and gizzard weights of quails from the various treatments were similar (*P* > 0.05).  However, the relative heart weight was higher in the control group than in the FBGP-supplemented groups (*P* < 0.05). 

**Table 3:** Effects of different levels of fermented black garlic powder supplementation on carcass traits and organ weight of male Japenese quails (*n* = 96)

Traits	Fermented black garlic powder levels, g/kg				SEM	Anova	Linear	Quadratic
	0	2.5	5.0	10.0				
HCW (g)	173.0	176.6	184.0	184.6	2.686	0.356	0.091	0.781
Heart (%)	1.18^a^	1.04^ab^	0.90^c^	0.99^b^	0.035	0.034	0.021	0.074
Liver (%)	2.39	2.12	2.09	1.98	0.061	0.095	0.021	0.454
Testis (%)	1.94	1.90	2.33	2.24	0.147	0.693	0.344	0.918
Gizzard (%)	1.83	1.83	1.86	1.61	0.051	0.320	0.186	0.251

^a, b^ Means with different superscripts in the same row were significantly different at *P* < 0.05. S.E.M.: Standard Error of the Mean, HCW: Hot

Carcass Weight

According to Table 4, the addition of FBGP to the diets of male quails did not affect AST, cholesterol and LDL values (*P>*0.05), while its effect on glucose, ALT, triglyceride and HDL values was found to be significant (*P* < 0.05). Serum glucose level decreased linearly with the FBGP supplementation from 359 mg/dL to 321 mg/dL. The serum triglyceride levels decreased linearly with the FBGP supplementation from 132.80 mg/dL to 98 mg/dL. Serum ALT levels were lower in the FBGP-supplemented groups compared to the control group, and this decrease was more pronounced at the 10.0 g/kg level. The serum HDL levels decreased with FBGP supplementation up to 5.0 g/kg, whereas they increased at 10.0 g/kg.

**Table 4:** Effects of different levels of fermented black garlic powder supplementation on some bloods traits of male Japanese quails (*n* = 96)

Traits	Fermented black garlic powder levels, g/kg				SEM	Anova	Linear	Quadratic
	0	2.5	5.0	10.0				
Glucose (mg/dl)	359.60^a^	355.20^b^	336.40^b^	321.00^b^	3.803	0.001	0.000	0.407
AST (U/L)	228.00	218.20	214.40	243.80	4.479	0.082	0.246	0.026
ALT (U/L)	16.14^a^	14.42^b^	13.86^b^	13.00^c^	0.258	0.000	0.000	0.052
Cholesterol (mg/dl)	177.00	170.00	169.20	184.80	4.356	0.127	0.163	0.059
Triglyceride (mg/dl)	132.80^a^	105.00^b^	101.20^b^	98.00^b^	3.236	0.000	0.000	0.001
HDL (mg/dl)	141.64^ab^	127.80^b^	131.04^b^	154.08^a^	3.519	0.023	0.146	0.006
LDL (mg/dl)	15.96	18.44	18.48	20.00	1.671	0.878	0.450	0.893

^a, b^ Means with different superscripts in the same row were significantly different at *P* < 0.05. S.E.M.: Standard Error of the Mean, AST : Aspartat Aminotransferaz, ALT : Alanin Aminotransferaz, HDL : High Density Lipoprotein, LDL: Low Density Lipoprotein

The effects of different levels of FBGP supplementation in male quail diets on immune traits are presented in Table 5. In this experiment, significant differences (*P <* 0.05) were observed for the IgE *(P <*0.00), IgA (*P <*0.00) and IgG (*P <*0.05) between experimental treatments. IgE levels decreased with the addition of FBGP to the diet but were similar among the FBGP supplemented groups. IgA levels increased linearly with the FBGP supplementation, and this increase was more pronounced at 5.0 and 10.0 g/kg. IgG levels were significantly higher in the FBGP-supplemented groups compared to the control group.

**Table 5:** Effects of different levels of fermented black garlic powder supplementation on immune traits of male Japanese quails (*n* = 96)

Traits	Fermented black garlic powder levels, g/kg				SEM	Anova	Linear	Quadratic
	0	2.5	5.0	10.0				
IgE (g/L)	10.22^a^	2.44^b^	2.26^b^	3.52^b^	0.718	0.000	0.000	0.000
IgA (g/L)	0.52^c^	0.61^b^	0.72^a^	0.73^a^	0.019	0.000	0.000	0.041
IgG (g/L)	0.22^b^	0.24^a^	0.25^a^	0.25^a^	0.004	0.045	0.014	0.142

^a, b^ Means with different superscripts in the same row were significantly different at *P* < 0.05. S.E.M.: Standard Error of the Mean, IgE: Immünoglobulin E, IgA: Immünoglobulin A, IgG: Immünoglobulin G

The effects of adding different levels of FBGP to growing quail diets on antioxidant parameters are shown in Table 6. Based on the experiment, no significant difference (*P* > 0.05) in antioxidant parameters was observed between the experimental treatments.

**Table 6:** Effects of different levels of fermented black garlic powder supplementation on antioxidant traits of male Japanese quails (*n* = 96)

Traits	Fermented black garlic powder levels, g/kg				SEM	Anova	Linear	Quadratic
	0	2.5	5.0	10.0				
TAS (mmol/L)	0.826	0.815	0.715	0.853	0.030	0.437	0.946	0.246
TOS (mmol/L)	3.064	2.826	2.351	2.799	0.108	0.126	0.178	0.107

S.E.M.: Standard Error of the Mean, TAS: Total antioxidant status, TOS: Total oxidant status

## Discussion

The primary goal of modern poultry farms is to increase nutrient utilization and growth rate with minimal feed intake (Ogbuewu et al. 2019). The increase in the use of plant-based feed additives in poultry diets in recent years has reduced producers’ dependence on commercial additives and is also considered a cost-effective alternative to synthetic growth promoters (Basit et al. 2025).

Increasing levels of FBGP added to the diet had no significant effect on quail performance parameters. In previous studies on this subject, Bayat and Petek (2024) found that 1% black garlic addition to broiler chicken diets did not affect performance parameters. Although studies on black garlic are limited in the literature, previous studies using white garlic, conducted in previous years, by Choi et al. (2010), reported that 1%, 3%, and 5% garlic powder addition to broiler chicken diets did not affect performance parameters. Eid and Iraqi (2014) reported that increasing amounts of garlic powder added to broiler chicken feeds increased performance parameters. They attributed this increase to higher feed efficiency. The current study is similar to Bayat and Petek (2024) and Choi et al. (2010), but contradicts Eid and Iraqi (2014). The reason for this difference may be due to the animal breed, diet and the trial period.

Carcass and organ parameters were not affected by the addition of black garlic powder to the diet except relative heart weight. In studies on the subject, Bayat and Petek (2024) reported that the addition of 1% black garlic powder to broiler chicken diets did not affect carcass parameters. Tanti et al. (2022) found that the addition of 3% black garlic powder to broiler chicken diets did not affect carcass parameters. Raeesi et al. (2010) found that the addition of 0.5, 1.0 and 3.0% garlic powder to broiler chicken diets reduced heart weight. Khatua et al. (2016) showed that diallyl disulfide, the active component of garlic, reduced isoproterenol-induced rat cardiac hypertrophy. The researchers suggested that this effect was due to the fact that the application of diallyl disulfide stimulated mitochondrial biogenesis by reducing oxidative stress and activating the eNOS–Nrf2–Tfam pathway; they reported that this resulted in a lower heart weight/body weight ratio. Based on this result, it can be said that the decrease in heart weight is due to a reduction in oxidative stress. In the current study, the relative decrease in heart weight is thought to be due to the oxidative stress-reducing effect of the powerful antioxidant compound S-allyl cysteine, found in FBGP.

In the current study, serum glucose concentration decreased significantly in the treatment groups compared to the control group (*P* < 0.01). This result may be due to the hypoglycemic and glucose metabolism regulating effects of FBGP. Singh et al. (2017) found that 1%, 1.5% and 2% garlic powder addition to broiler diets significantly reduced serum glucose levels. They reported that this decrease in serum glucose levels might be due to garlic’s enhancing effect on pancreatic insulin secretion from beta cells. Augusti and Sheela (1996) stated that the S-allyl cysteine ​​sulfoxide contained in garlic stimulates insulin secretion. The results of our study are in agreement Singh et al. (2017). A significant decrease in serum ALT concentration was observed in the treatment groups compared to the control group (*P* < 0.001). In a study on the subject, Fathi et al. (2025) stated that the addition of 10, 20, 30 and 40 g/kg of black garlic powder to broiler chicken diets reduced serum ALT levels. Additionally, Kairalla et al. (2022) reported that garlic supplementation to broiler chicken diets at 1%, 2%, and 3% caused a significant decrease in serum ALT levels. The results were similar, and the observed decrease in serum ALT concentrations could be attributed to the fact that FBGP reduces hepatic cell damage and improves liver function. Jimoh et al. (2012) reported that the hepatoprotective activity of garlic is due to its antioxidant effect. The addition of FBGP to the diet reduced serum triglyceride levels. Fathi et al. (2025) found that the addition of FBGP at 10, 20, 30, and 40 g/kg to broiler chicken diets reduced serum triglyceride levels. Berliana et al. (2021) emphasized that the addition of black garlic powder to broiler chicken diets at levels of up to 3% resulted in a significant decrease in triglyceride levels. The decrease in serum triglyceride levels may be due to an increase in pancreatic enzyme activities and enhanced conversion of fat to glycerol due to increased lipase secretion. The study results are in agreement with those of Berliana et al. (2021) and Fathi et al. (2025). Serum HDL levels decreased in quails supplemented with 5.0 FBGP and increased at 10.0 g/kg.  Kairalla et al. (2022) found that garlic supplementation at levels of 1%, 2%, and 3% to broiler chicken diets increased serum HDL levels. They suggested that this increase in serum HDL levels stems from garlic’s hypocholesterolemic and hypolipidemic mechanisms of action, which may lead to a decrease in the hepatic activity of cholesterologenic and lipogenic enzymes such as malic enzyme, fatty acid synthase, and glucose-6-phosphate dehydrogenase. Furthermore, Berliana et al. (2021) reported that black garlic increases serum HDL levels in broiler chickens, and that this effect may be due to its high vitamin C and B content. In the present study, serum HDL levels increased with FBGP supplementation at 10.0 g/kg. This finding is in agreement with the results of Kairalla et al. (2022) and Berliana et al. (2021), who reported that dietary supplementation with garlic and FBGP increased serum HDL concentrations in broilers. However, at the 5.0 g/kg FBGP inclusion level, our results are inconsistent with those reported by Kairalla et al. (2022) and Berliana et al. (2021).

In the intensive poultry farming industry, one way to protect against potential risks is through a strong immune system. Immune-supporting feed additives are currently attracting attention. Akan (2014) reported that the high antioxidant content of black garlic enhances immune system activity. In the current study, the addition of different levels of FBGP to male Japanese quail diets positively affected serum immunoglobulin levels (IgE, IgA, IgG). IgE levels were significantly reduced with the addition of FBGP at 2.5 and 5.0 g/kg. This result suggests that FBGP suppresses IgE production, which plays a role in allergic and inflammatory responses. The immune system produces antibodies against foreign substances entering the body. The type of these antibodies associated with allergies is IgE (immunoglobulin E). Black garlic has been reported to exhibit antiallergic effects, reducing IgE-related hypersensitivity reactions (Ergin 2019). In addition, it can be said that the anti-inflammatory and immunomodulatory properties of fermented black garlic may be due to the organosulfur compounds in garlic. Hsieh et al. (2019) investigated the effects of garlic extract (at doses of 100, 150, and 200 mg/kg) in mice using a Dermatophagoides pteronyssinus mite-induced allergic asthma model. As a result of the study, garlic extract significantly reduced serum IgE levels, which was interpreted as an indicator of the protective effect of garlic extract on allergic inflammation. Serum IgA levels increased significantly with the addition of FBGP (*P* < 0.001). Compared to the control group, serum IgA values ​​were significantly higher at 5.0 and 10.0 g/kg supplementation. A linear increasing trend indicates that IgA levels increased steadily with the dose. This observed increase in serum IgA levels may be due to the stimulatory effect of fermented black garlic on the mucosal immune response. Because IgA is the first line of defense against pathogens in the gastrointestinal and respiratory systems, this increase in serum IgA levels is thought to have a positive effect on the general immune status of male Japanese quails. Sözcü (2009) reported that the addition of 5 mg of garlic extract to broiler chicken drinking water had no significant effect on IgA concentration. Chang et al. (2021) reported that the addition of 0.06 mL/L garlic oil to laying hen drinking water increased serum IgA levels, attributing this to the immune system-enhancing properties of garlic essential oil. Yu et al. (2021) reported that the addition of 1000 and 2000 mg/kg garlic powder to broiler chicken diets increased serum IgA levels. The study results contradict Sözcü (2009) but are similar to Chang et al. (2021) and Yu et al. (2021). When the effects of FBGP supplementation on serum IgG levels in male Japanese quail were examined, IgG concentrations were found to increase significantly in all treatment groups compared to the control group. Fathi et al. (2025) found that adding different levels of black garlic powder to broiler chicken feed (10, 20, 30, and 40 g/kg) significantly increased serum IgG levels. Researchers have emphasized the potential of plant antioxidants added to the diet to strengthen humoral immune capacity by increasing serum immunoglobulin levels. El-Gogary et al. (2017) added garlic essential oil to growing rabbit diets at levels of 0.25, 0.50, and 0.75 g/kg. The study reported that adding garlic oil up to 0.50 g/kg increased serum IgG levels and decreased it at 0.75 g/kg levels. This was attributed to garlic oil’s ability to improve immune responses. In another study conducted on rabbits, Alagawany et al. (2016) reported that adding 6 g/kg of garlic powder to the diet increased serum IgG levels. The study results were similar to other researcher result.

In recent years, research on oxidative stress and antioxidant agents has become prominent (Aydogan et al. 2020). Black garlic contains powerful antioxidant compounds (Shahbazi et al. 2021). In the present study, the addition of FBGP as a feed additive at increasing levels to male Japanese quail diets did not cause an improvement in the oxidative status of the quails. Although TOS levels decreased numerically and TAS levels increased numerically at 10.0 g/kg compared to the control group, this did not constitute a significant difference. Our findings agree with the results of Aydogan et al. (2020) reported that 5 g/kg garlic supplementation to broiler diets did not affect serum TAS and TOS levels. 

## Conclusions

Adding FBGP to the diets of male Japanese quails did not show a positive effect on growth performance, antioxidant capacity, and carcass and organ characteristics, except for the relative heart weight. However, supplementation with FBGP led to a decrease in serum glucose, triglyceride, and alanine aminotransferase (ALT) concentrations. Furthermore, FBGP supplementation positively modulated immune responses by decreasing serum IgE levels while increasing IgA and IgG concentrations. These findings suggest that FBGP could be considered a potential immune-boosting feed additive in poultry nutrition, as it improves immunological parameters without negatively impacting performance or carcass and organ weights. 

## Supplementary Information

Below is the link to the electronic supplementary material.


Supplementary Material 1


## Data Availability

Preliminary findings from this study indicate that fermented garlic used in male quail diets has notable effects on immune responses and selected hematological parameters, necessitating further research to determine the optimal dosage. As a patent application is planned based on the forthcoming results, the confidentiality and commercial sensitivity of the data are of critical importance. Therefore, the research data are not publicly available. However, data may be shared upon reasonable, scientifically justified request and only under appropriate confidentiality conditions.

## Declarations

### Generative AI and AI-assisted technologies in the writing process

During the preparation of this work, the author used ChatGPT (OpenAI) to improve language clarity and to assist in rephrasing sections of the manuscript. After using this tool, the author carefully reviewed, edited, and verified all content to ensure accuracy and integrity. The author take full responsibility for the content of the publication.
