# Supplementary material for: Effects of Dietary Fermented Black Garlic Powder Supplementation on Performance, Carcass Traits, Serum Parameters, Immune Response and Antioxidant Status of Japanese Male Quails
Source: Trop Anim Health Prod. 2026 Feb 12;58(2):105. doi: 10.1007/s11250-026-04871-8 (PMC12901153; doi:10.1007/s11250-026-04871-8)
Supplement: Supplementary file 1 — Supplementary Material 1 [file 11250_2026_4871_MOESM1_ESM.docx]

Necati Esener BEng, MSc, PhD Konya

+90 332 355 12 90

Agricultural Genetic Engineer

# SUMMARY

Research professional with focus on conducting thorough literature reviews, designing experiments, and analysing data. Adept at collaborating with cross- functional teams and presenting findings in clear, concise reports. Strong organisational skills and keen eye for detail ensure high-quality outcomes in all research activities. Experienced Lecturer with focus on creating engaging and impactful learning environments. Proficient in developing and delivering innovative curriculum that enhances student understanding and fosters academic growth.

# EXPERIENCE

## Konya Food and Agriculture University

Lecturer *09/2024 - Present* Konya, TURKEY


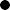
 Delivering undergraduate courses in statistics, biostatistics, data science.


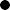
 Teaching precision agriculture, fostering the application of advanced technologies, and analytics in modern farming systems.


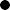
 Integrating theoretical knowledge with practical applications.

## Bahri Dagdas International Agricultural Research Institute

Head of International Projects Unit / Researcher

*03/2022 - Present*

Konya, TURKEY


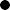
 Leading and contributing to interdisciplinary research projects focused on bioinformatics—including genomics, proteomics, and metagenomics—as well as the integration of artificial intelligence in agricultural science.


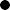
 Serving as the principal investigator on several nationally funded research projects, overseeing all stages from conception, design, implementation, and analysis.


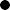
 Playing a central role in the statistical and methodological development of additional institutional research initiatives.


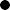
 Coordinating collaboration within major international funding frameworks such as Horizon Europe and COST.

[necati.esener@tarimorman.gov.tr](mailto:necati.esener@tarimorman.gov.tr)

# EDUCATION

04/2017 - 10/2021

University of Nottingham

UK

PhD: Veterinary, Medicine & Science

09/2015 - 09/2016

Aberystwyth University

UK

MSc: Livestock Science

09/2008 - 06/2013

Ege University

Turkey

Bachelor’s Degree:

Bioengineering

09/2011 - 02/2012

Politechnika Bydgoska

Poland

Erasmus Exchange

# CERTIFICATIONS

English YDS, 2022, Score: 95

English IELTS, 2015, Score: 6.5

# SKILLS


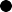
 Machine Learning


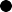
 Python Programming Language


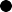
 R Programming Language


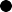
 Research and analysis
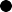
 Academic writing


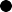
 Lecture planning


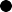
 Classroom management

# EXPERIENCE

## Ministry of Interior

Data Preparation and Control Operator

12*/2014 - 09/2015*

Ankara, TURKEY

## Alba Thyment Sp. z. o. o.

Intern (IAESTE)

*07/2012 - 08/2012*

Poznan, POLAND

# PUBLICATIONS

Articles Published in International Journals:


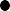
 Esener, N. (2025). Exploring trends in reproductive system microbiome research in farm animals: A bibliometric approach. Research in Veterinary Science, 186, 105583. <https://doi.org/10.1016/j.rvsc.2025.105583>.


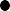
 Esener, N., Erduran, H., Keskin, İ. et al. Machine learning-based early prediction of growth and morphological traits at yearling age in pure and hybrid goat offspring. Trop Anim Health Prod 56, 262 (2024). <https://doi.org/10.1007/s11250-024-04145-1>.


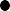
 Esener, N., Guerra, A. M., Giebel, K., Lea, D., Green, M. J., Bradley, A. J. and Dottorini, T. (2021) 'Mass spectrometry and machine learning for the accurate diagnosis of benzylpenicillin and multidrug resistance of Staphylococcus aureus in bovine mastitis', PLOS Computational Biology, 17(6), pp. e1009108. [https://doi.org/10.1371/journal.pcbi.1009108](https://doi.org/10.1007/s11250-024-04145-1).


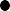
 Esener, N., Maciel-Guerra, A., Giebel, K., Lea, D., Green, M. J., Bradley, A. J. and Dottorini,

T. (2021) 'Prediction of Streptococcus uberis clinical mastitis treatment success in dairy herds by means of mass spectrometry and machine-learning', Scientific Reports, 11(1), pp. 7736. [https://doi.org/10.1038/s41598-021-87300-0.](https://doi.org/10.1038/s41598-021-87300-0)


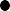
 Esener, N., Green, M. J., Emes, R. D., Jowett, B., Davies, P. L., Bradley, A. J. and Dottorini, T. (2018) 'Discrimination of contagious and environmental strains of Streptococcus uberis in dairy herds by means of mass spectrometry and machine-learning', Scientific Reports, 8(1), pp. 17517. [https://doi.org/10.1038/s41598-018-35867-6](https://doi.org/10.1038/s41598-021-87300-0).

International Seminars & Conferences attended as a speaker:


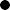
 2020 World Milk Day Conference (Oral Presentation), University of Nariño, Nariño, Colombia. 'Discrimination of contagious and environmental strains of Streptococcus uberis in dairy herds by means of mass spectrometry and machine-learning', Scientific Reports, 8(1), pp. 17517. [https://doi.org/10.1038/s41598-018-35867-6](https://doi.org/10.1038/s41598-021-87300-0).


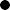
 2020 British Society of Animal Science (BSAS). 'Discrimination of contagious and environmental strains of Streptococcus uberis in dairy herds by means of mass spectrometry and machine-learning', Scientific Reports, 8(1), pp. 17517. [https://doi.org/10.1038/s41598-018-35867-6](https://doi.org/10.1038/s41598-021-87300-0).

2019 PGR Symposium (Oral Presentation), University of Nottingham, Nottingham, UK.’The Use of MALDI-TOF to Differentiate Phenotypic Profiles of Esherichia coli Isolates’.

2018 Genome Science Conference (Poster Presentation), University of Nottingham, Nottingham, UK. 'Discrimination of contagious and environmental strains of Streptococcus uberis in dairy herds by means of mass spectrometry and machine- learning', Scientific Reports, 8(1), pp. 17517. [https://doi.org/10.1038/s41598-018-35867-](https://doi.org/10.1038/s41598-021-87300-0) [6](https://doi.org/10.1038/s41598-021-87300-0).

2018 PGR Symposium (Oral Presentation), University of Nottingham, Nottingham, UK. ‘Implementation of machine learning for the evaluation of mastitis’.

Ph.D. Thesis, Master Thesis, Reports, Books etc.:


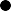
 Esener, Necati (2021). Implementation of machine learning for the evaluation of mastitis and antimicrobial resistance in dairy cows. PhD thesis, University of Nottingham. <http://eprints.nottingham.ac.uk/66056/>


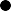
 Esener, Necati (2016). Identification of signatures of tissue-specific gene expression in Bos Taurus. Master thesis, Aberystwyth University.


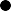
 Esener, Necati (2008). Ecotoxicology of human pharmaceuticals. Undergraduate thesis, Ege University.

# PROJECTS & GRANTS


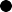
 Evaluation of Sheep Behavior with a Tri-Axial Accelerometer. (TUBITAK-1003, Project Leader, Project no: 123O716)


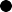
 Exploring the Relationship Between Vaginal Microbiota and Pregnancy in Central Anatolian Merino Ewes via 16S rRNA Sequencing (TUBITAK-1002, Project Leader, Project no: 124O668)


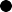
 Smart Flock Management Using Artificial Intelligence Technologies (TUBITAK-1711, Researcher, Project no: 3247006)


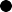
 Evaluation of Growth Performance, Carcass Characteristics, Meat Quality, and Fatty Acid Profile of Hasmer and Hasak Male Lambs Fed Under Different Fattening Systems (TAGEM Project, Researcher)


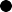
 Determination of Growth Curve Models for Lambs in Selected Indigenous Sheep Breeds (TAGEM Project, Researcher)

# ACADEMIC TEACHING ACTIVITIES


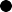
 Biostatistics (KGTU Undergraduate course, 2024-2025 Fall Semester)
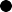
 Statistics (KGTU Undergraduate course, 2024-2025 Fall Semester)


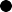
 Precision Agriculture (KGTU Undergraduate course, 2024-2025 Spring Semester)

# COURSES & CONFERENCES

Processing Technology of Agricultural and Livestock Products for Developing Countries, China, October 2023. Akitek Smart Agricultural Applications, Konya, Türkiye, August, 2023.

Training Program for Strengthening international Agricultural Cooperation, South Korea (online), October, 2022.
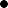
 8th International Cereal Nematodes Symposium, Bolu, Türkiye, September 2022


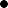
 Bacterial Genomes: Disease, Outbreaks and Antimicrobial Resistance, Welcome Sanger Institute, April, 2021.
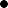
 Bacterial Genomes: Welcome Sanger Institute, Bacterial Genomes: Disease, Outbreaks and Antimicrobial

Resistance, April, 2021.


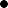
 Welcome Sanger Institute, Bacterial Genomes: Comparative Genomics using Artemis Comparison Tool (ACT), April, 2021.


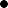
 Welcome Sanger Institute, Bacterial Genomes: From DNA to Protein Function Using Bioinformatics, April, 2021.
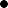
 Welcome Sanger Institute, Bacterial Genomes: Antimicrobial Resistance in Bacterial Pathogens, April, 2021.


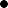
 British Society of Animal Science 2021 Annual Conference, April, 2021.


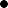
 Bacterial Genomes: From DNA to Protein Function Using Bioinformatics, Welcome Sanger Institute, April 2021.
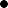
 Bacterial Genomes: Antimicrobial Resistance in Bacterial Pathogens, Welcome Sanger Institute, April 2021


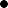
 British Society of Animal Science 2021 Annual Conference, April 2021.


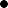
 Global Antibiotic Research & Development Partnership, Discovery of new antimicrobials using artificial intelligence (computational chemoinformatics), November, 2020.


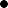
 Nature Masterclasses, Scientific Writing and Publishing, November, 2020.


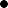
 Global Antibiotic Research & Development Partnership, Building better breakpoints: data and methods needed to determine breakpoints for new agents, October, 2020.


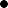
 Center for English Language Education, Academic Vocabulary Skills, October, 2020.
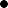
 Bionformatics from Algorithms to Applications Conference, August 2020.


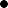
 University of Cambridge, An Introduction to Machine Learning, June, 2020.


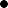
 NCBI Webinars, NCBI’s ALFA: allele frequency data for variant analysis and interpretation, April, 2020.
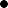
 University of Nottingham, Introduction to MATLAB for Engineers, March, 2020.


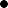
 University of Nottingham, Academic Writing Workshops: Managing and Citing References, February, 2020.
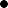
 University of Nottingham, Academic Writing Workshops: Reviewing Literature, February, 2020.


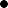
 University of Nottingham, UK Data Service Event, January, 2020.


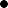
 Discovery of new antimicrobials using artificial intelligence (computational chemoinformatics), Global Antibiotic Research & Development Partnership, November 2020.


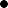
 Scientific Writing and Publishing, Nature Masterclasses, November 2020.


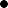
 Building better breakpoints: data and methods needed to determine breakpoints for new agents, Global Antibiotic Research & Development Partnership, October 2020.


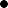
 Academic Vocabulary Skills, Center for English Language Education, October 2020.
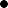
 Bionformatics from Algorithms to Applications Conference, August 2020.


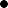
 An Introduction to Machine Learning, University of Cambridge, June 2020.


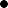
 NCBI’s ALFA: allele frequency data for variant analysis and interpretation, NCBI Webinars, April 2020.
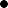
 Introduction to MATLAB for Engineers, University of Nottingham, March 2020.


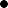
 Academic Writing Workshops: Managing and Citing References, University of Nottingham, February 2020.
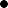
 Academic Writing Workshops: Reviewing Literature, University of Nottingham, February 2020.


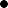
 UK Data Service Event, University of Nottingham, January, 2020.


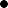
 Academic Writing Workshops: Managing and Citing References, University of Nottingham, February 2020.
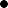
 Academic Writing Workshops: Reviewing Literature, University of Nottingham, February 2020.


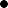
 UK Data Service Event, University of Nottingham, January, 2020.
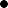
 University of Dundee, 3D RNA-seq Application, November, 2019.


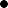
 The James Hutton Institute, Accurate and fast quantifation of transcriptomes using RNA-seq, November, 2019.
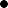
 University of Nottingham, Statistics and Design for Bioscientists, September, 2019.


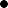
 University of Nottingham, Post-genomics and Bioinformatics, May, 2019.
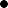
 3D RNA-seq Application, University of Dundee, November 2019.


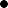
 Accurate and fast quantification of transcriptomes using RNA-seq, The James Hutton Institute, November 2019.
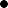
 Statistics and Design for Bioscientists, University of Nottingham, September 2019.


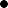
 Post-genomics and Bioinformatics, University of Nottingham, May 2019.


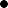
 University of East Anglia, Genome 10K and Genome Science Conference, August, 2017.
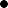
 University of Cambridge, An Introduction to MATLAB for biologists, May, 2017.


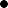
 Genome 10K and Genome Science Conference, University of East Anglia, August 2017.
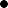
 An Introduction to MATLAB for biologists, University of Cambridge, May 2017.


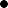
 British Society of Animal Science for a Sustainable Future Annual Conference. Genomics using Artemis Comparison Tool (ACT), 2021-04-01, Welcome Sanger Institute, April, 2016.


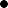
 British Society of Animal Science for a Sustainable Future Annual Conference, April 2016.
